# The p38 MAPK/PMK-1 Pathway Is Required for Resistance to *Nocardia farcinica* Infection in *Caenorhabditis elegance*

**DOI:** 10.3390/pathogens11101071

**Published:** 2022-09-21

**Authors:** Ruiqiu Yang, Yingqian Kang, Jiahong Duan, Chenggang Zou, Qinyi Wu

**Affiliations:** 1 State Key Laboratory for Conservation and Utilization of Bio-Resource in Yunnan, Yunnan University, Kunming 650091, China; 2 School of Basic Medical Sciences, Guizhou Medical University, Guiyang 550025, China; 3 Yunnan Provincial Key Laboratory of Molecular Biology for Sinomedicine, Yunnan University of Chinese Medicine, Kunming 650500, China

**Keywords:** *C. elegans*, *Nocardia farcinica*, p38 MAPK signaling, immune integrity, SKN-1

## Abstract

*Nocardia farcinica* is an opportunistic pathogen that causes nocardiosis primarily in patients with compromised immune systems. In this study, we used the genetically tractable organism *Caenorhabditis elegans* as a model to study the innate immune responses to *N. farcinica* infection. We found that unlike other pathogenic bacteria such as *Pseudomonas aeruginosa* and *Staphylococcus aureus*, *N. farcinica* failed to kill adult worms. In another words, adult worms exposed to *N. farcinica* exhibited a normal lifespan, compared with those fed the standard laboratory food bacterium *Escherichia coli* OP50. Interestingly, deletion of three core genes (*pmk-1*, *nsy-1* and *sek-1*) in the p38 MAPK/PMK-1 pathway reduced the survival of worm exposure to *N. farcinica*, highlighting a crucial role of this pathway for *C. elegans* in resistance to *N. farcinica*. Furthermore, our results revealed that *N. farcinica* exposure up-regulated the level of PMK-1 phosphorylation. The activation of PMK-1 promoted nuclear translocation of a transcription factor SKN-1/Nrf2, which in turn mediated *N. farcinica* infection resistance in *C. elegans*. Our results provide an excellent example that the integrity of immune system is key aspect for counteract with pathogenesis of *N. farcinica*.

## 1. Introduction

*Nocardia farcinica*, is an opportunistic pathogen under the genus of Actinomycetes widely distributed in the soil and rotten substances, causing lung infection through inhalation of bacterial particles [1]. Since the first case of pulmonary nocardiosis was reported in the USA in 1898, thousands of cases of pulmonary nocardiosis have been reported annually, primarily in patients with compromised immune systems [2]. Nocardia not only cause lung infections, but also erode other organs by entering blood vessels, causing localized or systematical infection in central nervous system, skin, subcutaneous tissue, retina, kidney, joint, bone, and heart [1]. Nocardia are also associated with Parkinson’s syndrome, as subcutaneous injection of Nocardia induce symptoms similar to Parkinson’s syndrome clinically and pathologically in mice [3]. A recent study reveals that Nocobactin NA-a and Nocobactin NA-b, two compounds isolated from *N. farcinica*, suppress immune responses by inhibiting Notch signal pathway in RAW264.7 macrophages [4]. Despite several studies have revealed the virulence of *N. farcinica* in mammal. The mechanism of pathogenesis in *N. farcinica* infection is not fully understood.

*Caenorhabditis elegans* is a free-living, bacterivorous nematode that lives in the soil, well known as invertebrate without adaptive immune system as well as specific immune cells [5]. In wild worms, a large microbial fauna is observed in the gut [6]. In the natural environment, *C. elegans* often encounters a variety of pathogens, thus it exerts a strong selective pressure to evolve and develop an effective innate immune system. *C. elegans* therefore represents a powerful invertebrate model to study the innate immune responses to nosocomial bacterial pathogens such as *Pseudomonas aeruginosa*, *Staphylococcus aureus*, *Salmonella enterica*, *Salmonella typhimurium*, and *Serratia marcescens* [7,8,9,10], and fungal species such as *Cryptococcus neoformans* [11] and *Candida albicans* [12].

During the last two decade, studies using *C. elegans* have revealed a variety of the evolutionarily conserved signaling pathways in the innate immunity, such as the DAF-2 insulin-like signaling pathway [13], the p38 mitogen-activated protein kinase (MAPK) pathway [14], and the ERK MAPK pathway [15], the JNK MAPK pathway [16], the protein kinase D pathway [17], the G protein coupled receptor FSHR-1 pathway [18], the G protein EGL-30 pathway [19], and the transcription factor TFEB pathway [20]. Of these signaling pathways, the p38 MAPK/PMK-1 pathway, which consists of a core cassette of NSY-1/MAPKKK, SEK-1/MAPKK, and PMK-1/MAPK, act in the intestine of *C. elegans* to regulate innate immunity [14,21]. PMK-1 mediates the expression of a set of immune-related genes, such as C-type lectins, ShK toxins, lysozymes, and antimicrobial peptides, which are involved in fighting off infection [22]. Meanwhile, a couple of the downstream transcription factors of PMK-1 that promote pathogen resistance have been identified. For instance, PMK-1 activates the IRE-1–XBP-1-dependent unfolded protein response (UPR) in *C. elegans* upon *P. aeruginosa* infection [23]. Interestingly, the role for XBP-1-mediated UPR is to protect worms against the lethal endoplasmic reticulum stress during activation of innate immunity. Furthermore, the transcription factor SKN-1 is activated by PMK-1, thereby conferring resistance *P. aeruginosa* infection [24]. Under normal conditions, the transcription factor ATF-7 inhibits PMK-1 regulated immune-related gene expression. However, the activation of PMK-1 by bacterial infection can phosphorylates ATF-7, which switches the transcription factor from a repressor to an activator of PMK-1–dependent immune gene expression [25].

In this study, we screened the major signaling pathways that are involved in defense against *N. farcinica* in *C. elegans*. We discovered that the PMK-1/p38 MAPK signaling was required for the survival of worms upon *N. farcinica* exposure. Further studies indicated that PMK-1/p38 MAPK activated the transcription factor SKN-1, which in turn conferred resistance to *N. farcinica* infection in *C. elegans*.

## 2. Results

### 2.1. The Lifespan of C. elegans Is Unaffected when Fed on N. farcinica

**Figure 1:**
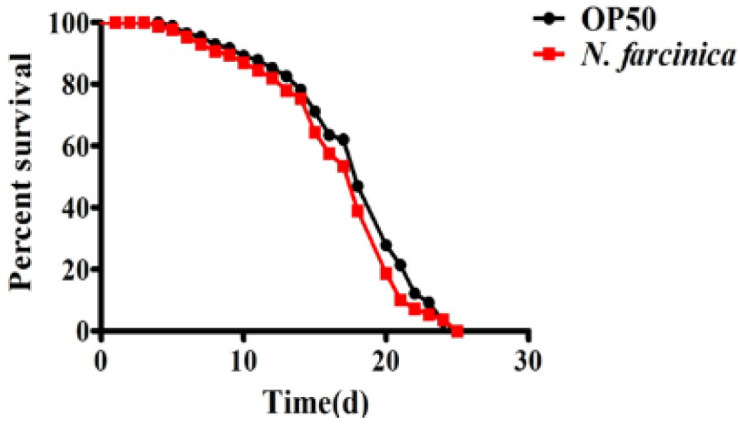
*N. farcinica* does not affect lifespan of adult *C. elegans*. Adult *C. elegans* was placed on BHI medium with either *E. coli* OP50 or *N. farcinica* at 25 °C, the lifespan was monitored at 24 h interval. The lifespan of WT worms was comparable when fed on *E. coli* OP50 and *N. farcinica*, respectively (log-rank *p* *=* 0.6). See Table S1 for details.

We first investigated the pathogenicity of *N. farcinica* in nematodes. However, we found that unlike worms exposed to *P. aeruginosa*, *S. aureus,* and *E. faecium* [26,27], wild-type (WT) worms exposed to *N. farcinica* exhibited a similar lifespan as those fed *E. coli* OP50, the standard laboratory food (Figure 1). Thus, *C. elegans* feeding on *N. farcinica* exhibits a normal lifespan.

### 2.2. The p38 MAPK Pathway Is Critical for C. elegans to Resist N. farcinica

**Figure 2:**
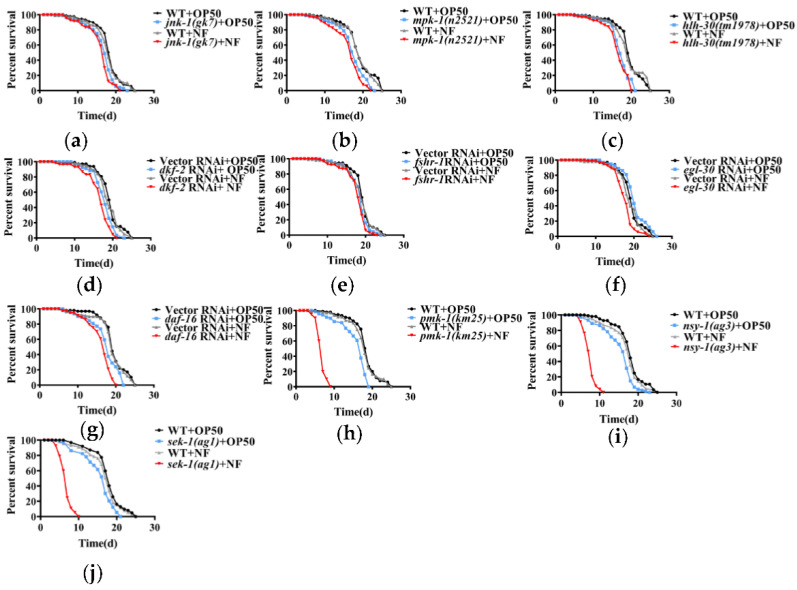
The role of the innate immune pathways in resistance to *N. farcinica* in worms. (**a**–**j**) Survival of *C. elegans* fed either *N. farcinica* (NF) or *E. coli* OP50 were monitored over time at 25 °C. The survival rates of *jnk-1(gk7)* (**a**), *mpk-1(n2521)* (**b**), *hlh-30(tm1978)* (**c**) mutants were comparable to worms in the presence of either *E. coli* OP50 or *N. farcinica*. RNAi knockdown of *dkf-2* (**d**), *fshr-1* (**e**), *egl-30* (**f**), *daf-16* (**g**) did not influence resistance to *N. farcinica*. Mutations in *pmk-1(km25)* (**h**), *nsy-1(ag3)* (**i**), *sek-1(ag1)* (**j**) enhanced susceptibility of worms to *N. farcinica*, (log-rank *p* < 0.001). See Table S1 for details.

Although *C. elegans* does not have a canonical adaptive immune system, it has evolved an innate immune mechanism to counteract bacterial infection. However, as a bacterial pathogen, *N. farcinica* had no effect on lifespan in worms. The fact that *N. farcinica* mainly infects immunoincompetent patients prompted a hypothesis that *C. elegans* relies on its innate immune defenses against *N. farcinica* infection. To verify this hypothesis, we screened the major innate immune pathways in worms, such as ERK MAPK/MPK-1 [28], JNK MAPK/JNK-1 [16], p38 MAPK/PMK-1 [14], DAF-2/DAF-16 [13], G protein coupled receptor FSHR-1 [18], protein kinase PKD/DKF-2 [17], transcription factor TFEB/HLH-30 [20], and G protein EGL-30 [19]. We found that *mpk-1, jnk-1*, *hlh-30* mutant strains which fed on either *E. coli* OP50 or *N. farcinica* had comparable lifespans (Figure 2a–c). Meanwhile, RNAi knockdown of *fshr-1*, *dkf-2*, *egl-30* or *daf-16* failed to affect lifespans when exposed to either *E. coli* OP50 or *N. farcinica* (Figure 2d–g). However, we observed slight lifespan shortness in mutant strains and RNAi knockdown worms compared to WT strains or subjected to empty vector RNAi, respectively (*p* ≤ 0.01). In contrast, *pmk-1(km25)* mutants exhibited marked decrease in lifespan when exposed to *N. farcinica*, compared to worms fed on *E. coli* OP50 and WT (Figure 2h). NSY-1/MAPKKK and SEK-1/MAPKK are kinases located upstream of PMK-1/p38 MAPK, which are involved in its activation through phosphorylation [14]. In the current study, the *nsy-1(ag3)* and *sek-1(ag1)* mutants exposed to *N. farcinica* also showed a significant reduction in lifespan, compared to *E. coli* OP50 diet and WT (Figure 2i,j). These results suggested that the p38 MAPK pathway is essential for survival of worms exposed to *N. farcinica*. 

### 2.3. C. elegans Depended on PMK-1 Activation to Resist N. farcinica Infection

**Figure 3:**
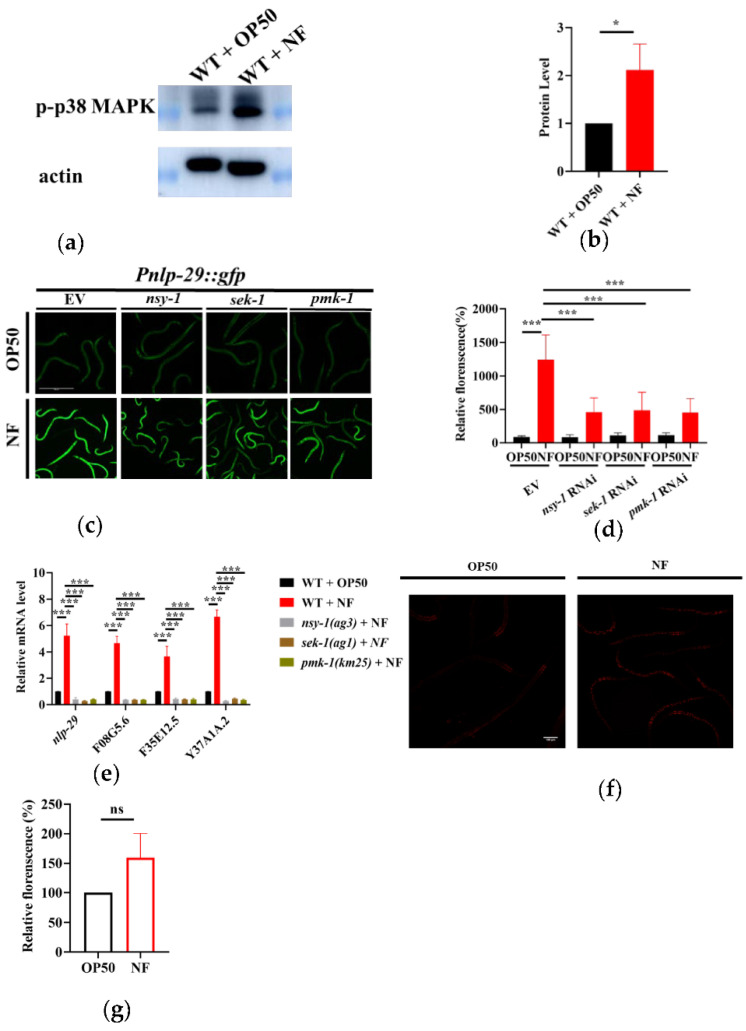
*N. farcinica* activates the p38 MAPK pathway in adult worms. (**a**) The phosphorylation of p38 MAPK was increase after feeding on *N. farcinica* (NF) for 24 h. (**b**) The level of phosphorylated p38 MAPK were quantified using Image J. The result is analyzed by t-test. * *p* < 0.05 versus *E. coli* OP50. (**c**) The expression of *Pnlp-29::gfp* was up-regulated in WT worms, but weaker in worms subjected to *nsy-1*, *sek-1* and *pmk-1* RNAi exposed to *N.farcinica*. Scale bar, 500 µm. (**d**) The fluorescent intensity of *Pnlp-29::gfp*. These results are means ± SD of three independent experiments (*n* = 35 worms per experiment). *p* values were calculated by one-way ANOVA followed by a Student-Newman-Keuls test and t-test. *** *p* < 0.001 versus *E. coli* OP50. (**e**) The mRNA levels of PMK-1 target genes (*nlp-29*, *F08G5.6*, *F35E12.5* and *Y37A1A.2*) after feeding on either *E. coli* OP50 or *N. farcinica*, in WT or *nsy-1*(*ag3*), *sek-1(ag1)*, or *pmk-1*(*km25*). *** *p* < 0.001 versus wild type. (**f**) DHE stain showed that *N. farcinica* induced slightly growing level of ROS in worms after 24 h exposure. Scale bar, 100 µm. (**g**) Quantification of relative fluorescent intensity of the DHE stain. *n* = 40, ns, not significant.

We then asked if *N. farcinica* activated PMK-1 by determining the phosphorylation level of PMK-1. We found that the phosphorylation levels of PMK-1 were significantly increased in worms exposed to *N. farcinica* for 24 hours, compared to those fed *E. coli* OP50 (Figure 3a,b). The increase in phosphorylation level of PMK-1 has also been reported when *C. elegans* was challenged by pathogens including *Pseudomonas aeruginosa* PA14 [29] and *Cutibacterium acnes* [30]. A previous study has demonstrated that the up-regulation of antimicrobial peptide gene *nlp-29* is mediated by PMK-1 [31]. We thus asked if PMK-1 activation upon exposure to *N. farcinica* can up-regulate the *nlp-29* expression (Figure 3c,d). As *F08G5.6*, *F35E12.5* and *Y37A1A.2* have been reported as PMK-1-regulated genes [22], we then verified the mRNA levels of these genes after *N. farcinica* exposure. The expressions of *F08G5.6*, *F35E12.5* and *Y37A1A.2* were significantly up-regulated after exposure to *N. farcinica*. In contrast, mutations in *nsy-1(ag3)*, *sek-1(ag1)*, and *pmk-1(km25)* suppressed their mRNA levels (Figure 3e). Taken together, these results suggested that *N. farcinica* exposure activates the p38 MAPK pathway in worms. In previous study, several gens like alkylhydroperoxide reductase (ahpC), catalase (katG), and superoxide dismutase (sodF) are predicted with high expressivity in *N. farcinica*, which could defense against reactive oxygen species (ROS) [32]. We thus checked the ROS level in worms through DHE stain. We found that the level of ROS was only increased slightly after *N. farcinica* infection. Thus, ROS are unlikely to be involved in the activation of the p38 MAPK pathway in worms.

### 2.4. N. farcinica Exposure Activates the Transcription Factor SKN-1 in a p38 MAPK Dependent Manner

**Figure 4:**
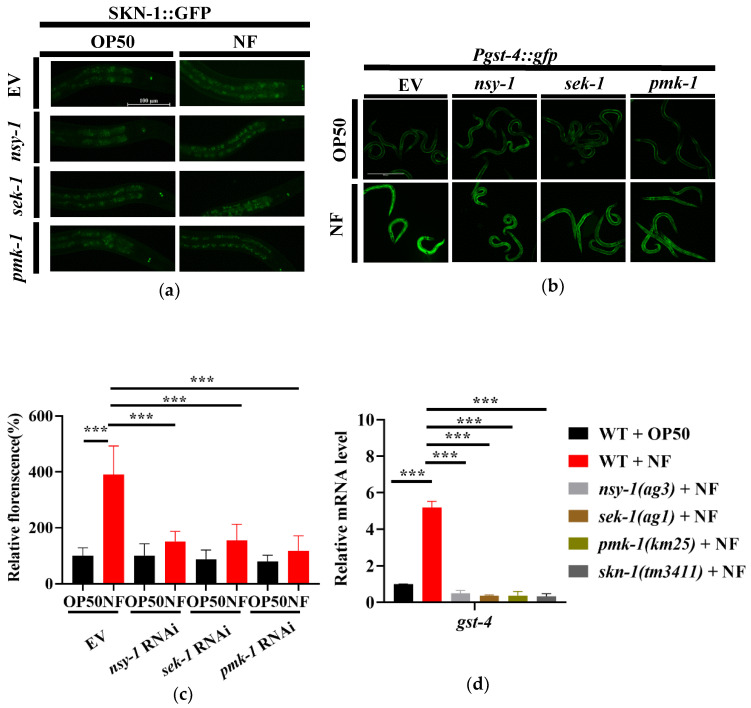
*N. farcinica* activates SKN-1 through the p38 MAPK pathway. (**a**) The nuclear translocation *of* SKN-1::GFP was increased in worms fed *N. farcinica* for 24 h. Scale bar, 100 µm. (**b**) The expression of *Pgst-4::gfp* in worms in the presence of *E. coli* OP50 or *N. faricinica* for 24 h. Scale bar, 500 µm. (**c**) Quantification of *Pgst-4::gfp* fluorescence intensity in worms. These results are means ± SD of three independent experiments (*n* = 40 worms per experiment). *p* values were calculated by one-way ANOVA followed by a Student-Newman-Keuls test and t-test. *n* = 40, *** *p* < 0.001. (**d**) qPCR of *gst-4* mRNA level in worms exposed to either OP50 or *N. farcinica* for 24 h. *** *p* < 0.001.

The transcription factor SKN-1/Nrf2 is a key downstream molecule of the p38 MAPK pathway [33]. PMK-1 has been reported to activate SKN-1 under both oxidative stress [34] and pathogenic infection conditions [24]. In *C. elegans*, SKN-1 confers resistance to Gram-negative bacteria *P. aeruginosa* PA14 and Gram-positive bacteria *E. faecalis* [33]. To determine whether SKN-1 was activated after *N. farcinica* infection, we used transgenic worms expressing SKN-1b/c::GFP+rol-6 to detect the nuclear translocation of SKN-1, which is an indicator of its activation [35]. Under standard growth conditions, SKN-1 was mainly located in the cytoplasm in all tissues (Figure 4a). By contrast, the SKN-1 nuclear translocation was observed in the nucleus of intestine upon 24 hours of *N. farcinica* exposure. However, knockdown of *nsy-1*, *sek-1* or *pmk-1* by RNAi significantly reduced the nuclear translocation of SKN-1 in worms exposed to *N. farcinica* (Figure 4a). The second phase detoxification gene *gst-4* (encoding Glutathione S-transferase-4) is a target gene of SKN-1 [36,37]. We thus used transgenic worms expressing the *Pgst-4::gfp* reporter gene to further demonstrate the activation of SKN-1 by *N. farcinica* infection. We found that the expression level of *Pgst-4::gfp* was dramatically increased in worms exposed to *N. farcinica* for 24 hours, The increase is abolished when worms subjected to *skn-1*, *nsy-1*, *sek-1* or *pmk-1* RNAi (Figure 4b,c). qPCR analysis showed that mutations in *skn-1(tm3411)*, *nsy-1(ag3)*, *sek-1(ag1),* and *pmk-1(km25)* suppressed up-regulation of *gst-4* expression induced by *N. farcinica* infection (Figure 4d). Taken together, our study indicated that *N. farcinica* infection activates SKN-1via the p38 MAPK pathway.

### 2.5. SKN-1 Confers Resistance to N. farcinica Infection

**Figure 5:**
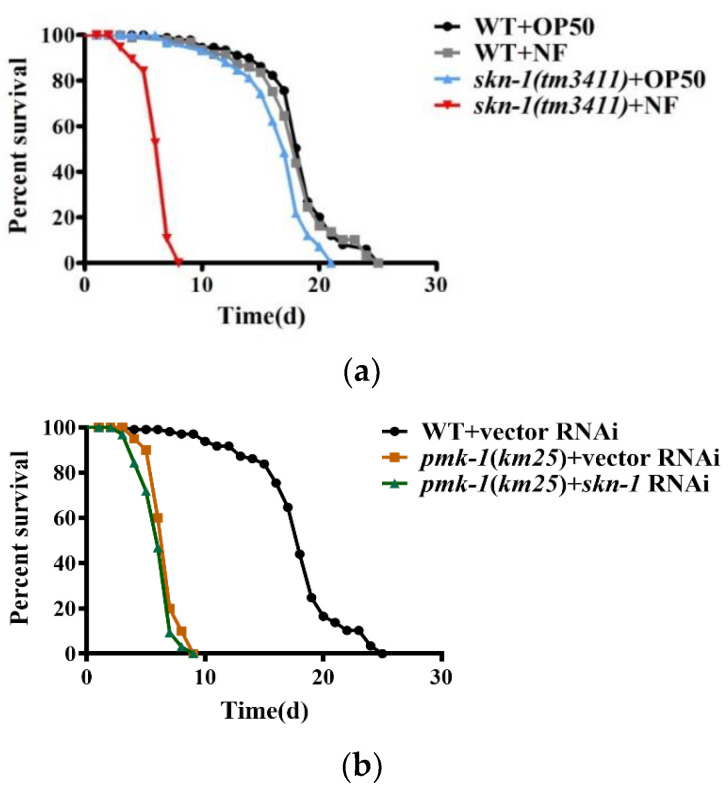
SKN-1 promotes worm resistance to *N. farcinica.* (**a**) Survival of wild type (WT) worms and *skn-1(tm3411)* mutants fed either *E. coli* OP50 or *N. farcinica* at 25 °C. *skn-1(tm3411)* worms were more sensitive to *N. farcinica* than WT worms. *p* < 0.001. (**b**) The susceptibility of *pmk-1(km25)* worms subjected to empty vector was similar to that of *pmk-1(km25)* worms subjected to *skn-1* RNAi in the presence of *N. farcinica*, (log-rank *p* = 0.15).

*skn-1* RNAi, indicating that *skn-1* is required to resist this pathogen. However, the survival rate of the *pmk-1(km25)* mutants was comparable to that of the *pmk-1(km25)* mutant subjected to *skn-1* RNAi upon *N. farcinica* infection (Figure 5b), indicating that *skn-1* is epistatic to *pmk-1*. Taken together, these results suggest that the p38 MAPK pathway mediates resistance to *N. farcinica* infection by activating SKN-1.

### 2.6. PMK-1 and SKN-1 Are Critical in Elimination of N. farcinica

**Figure 6:**
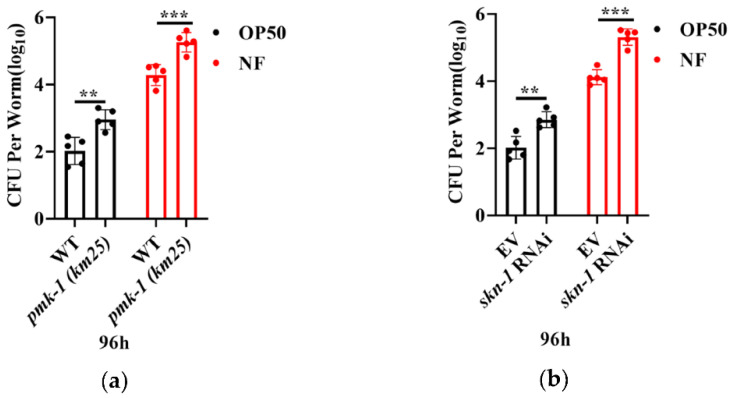
PMK-1 and SKN-1 are critical in elimination of *N. farcinica*. The colony forming units (CFU) of *N. farcinica* in young adult worms exposed to either *E. coli* OP50 or *N. farcinica* for 96 h. (**a**) *pmk-1(km25)* worms showed an increase in CFU of either *E. coli* OP50 or *N. farcinica*. ** *p* < 0.01 versus wild type, *** *p* < 0.001 versus wild type. (**b**) Inhibition of *skn-1* by RNAi increased CFU of these two bacteria in worms. ** *p* < 0.01 versus vehicle, *** *p* < 0.001 versus vehicle. For each group, 50–60 worms were used in single measurement, and 6–10 independent experiments were carried out, *p* values were calculated using *t*-test.

The successful colonization of bacteria is known as a key step in conducing infection. To ask if *N. farcinica* colonized in worms, young adult worms were exposed with either *E. coli* OP50 or *N. farcinica* on BHI plates for 96 hours. We found that RNAi knockdown of *pmk-1* and *skn-1* significantly increased the colony forming units (CFU) of *N. farcinica* and *E. coli* OP50 in the body of worms (Figure 6a,b). These results suggest that PMK-1 and SKN-1 are required for limitation of bacterial accumulation in the intestine of worms.

### 2.7. PMK-1 and SKN-1 Protects C. elegans from Tissue Damage upon N. farcinica Infection

**Figure 7:**
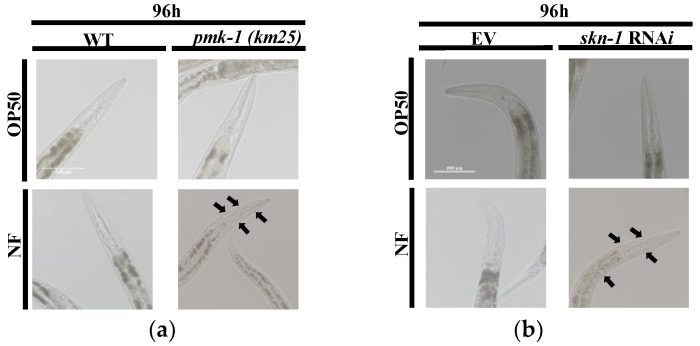
PMK-1 and SKN-1 prevent tissus damages upon *N. farcinica* exposure. DIC images of necrotic cells found in head of worms after 4 days of infection. (**a**) *pmk-1(km25)* worms showed enlarged vacuoles in the head of worms after *N. farcinica* infection. (**b**) Worms subjected to *skn-1* RNAi exhibited enlarged vacuoles in the head of worms. Enlarged vacuoles are pointed out by black arrows. Scale bar, 100 µm.

To determine whether *N. farcinica* induced tissue damage after long time infection in the worms, we observed the DIC images of *pmk-1 (km25)* worms, *skn-1* (RNAi) worms, and control group worms. As black arrows pointing out, genetic inactivation of *pmk-1* and *skn-1* caused necrosis in the head of worms exposed to *N. farcinica*, but not to *E. coli* OP50 (Figure 7a,b). These results suggest that PMK-1 and SKN-1 plays a protective role in tissue damages resulting from *N. farcinica* infection. 

## 3. Discussion

Our study demonstrated that the PMK-1/p38 MAPK pathway confers resistance to *N. farcinica* infection in *C. elegans*. This pathway is identified by screening eight signaling pathways required for innate immune responses to bacterial infections. Furthermore, our results indicated that *N. farcinica* exposure mediated the phosphorylation of p38 MAPK increased, which in turn activates SKN-1/Nrf2. The transcription factor is involved in *C. elegans* defense against *N. farcinica*.

In *C. elegans*, the PMK-1/p38 MAPK pathway plays a central role in innate immune response to infection with pathogenic bacteria, such as *P. aeruginosa* [14,38] *S. aureus* [39], *Salmonella* [7], *Yersinia pestis* [40], and *Mycobacteria* [41]. The p38 MAPK pathway mediates the resistance to bacterial infection and bacterial toxins, such as the toxin Cry5B [16] secreted by *Bacillus thuringiensis* and Shiga-like toxin of enterohemorrhagic *E. coli* O157:H7 [42]. In mammals, the p38 MAPK pathway is also involved in defense against pathogens [43,44], suggesting that this pathway may represent an ancient feature of metazoan innate immunity. Interestingly, *N. farcinica* can activate dendritic cells via the p38 MAPK pathway, leading to release of interleukin-12 (IL-12/23p40), known as key immune factor, mediates resistance to Mycobacterium. spp [45,46]. These results implicate that p38 MAPK is a conserved pathway mediates host resistance to *N. farcinica* in organisms ranging from *C. elegans* to mammals.

As a transcription factor, SKN-1 is involved in resistance to oxidative stress by upregulating a set of the Phase II detoxification genes [37]. SKN-1 also confers resistance to infections with pathogenic bacteria, such as *P. aeruginosa*, *E. faecalis* [24], Mycobacterium [41], and *Aeromonas dhakensis* [47]. In the current study, SKN-1/Nrf2 is required for innate immune responses to *N. farcinica* infection. It has been reported that PMK-1 can phosphorylate SKN-1 at Ser-74 and Ser-340 [34]. The phosphorylation of SKN-1 promotes its nuclear translocation and activation. Like bacterial pathogens including *P. aeruginosa*, *E. faecalis* and *N. farcinica* infection activates SKN-1 in a p38 MAPK-dependent manner. These results suggest that SKN-1 is one of downstream effectors of p38 MAPK that mediates *C. elegans* innate immune responses to both Gram-negative and Gram-positive pathogens. The molecular mechanism underlying SKN-1-mediated innate immunity remains unclear. Previously, we and others have demonstrated that autophagy plays an important role in *C. elegans* defense against a variety of pathogenic bacteria by repairing organismal insults [15,20,48]. SKN-1 up-regulates a set of autophagic genes in the germline-deficient *glp-1*(e2141ts) mutants [49]. Meanwhile, SKN-1 is involved in activation of autophagy in long-lived *hyl-1*; *lagr-1* mutants [50]. Thus, SKN-1 is likely to limit the damage induced by *N. farcinica* infection via promoting autophagy. The opportunistic infections followed immunologic deficiency have mainly been seen in HIV infected patients, revealing that the environmental “harmless” microorganisms could be life threaten, such as *Penicillium marneffei*, known as the third common opportunistic pathogen that causes penicilliosis in HIV patients. Likewise, *N. farcinica* is an opportunistic pathogen, with the majority of infections occurring in immunocompromised patients. In the current study, WT worms do not have a reduced lifespan when exposed to *N. farcinica*. These data suggested that like humans, WT worms with an intact innate immune system can resist to *N. farcinica* infection, but lacking key genes in the PMK-1/p38 MAPK pathway become susceptible to *N. farcinica* infection. In our studies, we also noticed that lack of p38 MAPK pathway and SKN-1 leads to significant increases accumulation of *N. farcinica* and obvious enlarged vacuoles in the head of worms. Thus, our finding emphasizes that the integrity of immune system is crucial for defense against *N. farcinica* infection in *C. elegans*.

What are the critical signal molecules from *N. farcinica* to activate the p38 MAPK/PMK-1 pathway?However, our data demonstrate that ROS are not the signal component produced by *N. farcinica*. Several virulence factors of *N. farcinica* have been reported when the pathogen infected mammals. For instance, Nfa34810 protein of *N. farcinica* can stimulate macrophages to produce tumor necrosis factor alpha (TNF-α) and other immune related factors through the TLR4 pathway [51]. Besides, *N. farcinica* IFM 11523, which isolated from Japanese patient, secretes two pathogenesis factors nocobactin NA-a (compound 1) and nocobactin NA-b (compound 2), which act as on the notch signaling inhibitors [4]. Thus, the signal molecules that activate the p38 MAPK/PMK-1 pathway need be investigated further in light of our present study.

## 4. Materials and Methods

### 4.1. C. elegans and Bacteria Strains

The *C. elegans* strains were maintained on NGM medium containing *E. coli* OP50 under standard condition. Strain used in this study include *C. elegans* Bristol N2, *pmk-1(km25)*, *mpk-1(n2521)*, *let-60(ga89)*, *jnk-1(gk7)*, *hlh-30(tm1978)*, *skn-1(tm3411)*, *nsy-1(ag3)*, *sek-1(ag1)*, *skn-1b/c::gfp+rol-6(su1006)*, *nlp-29p::gfp+col-12p::DsRed*, *gst-4p::gfp::NLS(pAF15)* were kindly provided by the Caenorhabditis Genetics Center (CGC), which is funded by NIH Office of Research Infrastructure Programs (P40 OD010440). Bristol N_2_ strain of *C. elegans* is used as non-genetic modified control in experiments, and labeled as wild-type (WT) in our study [52,53].

### 4.2. RNA Interference

Bacterial strains contain RNAi targeting genes were obtained from Ahringer library [54]. *E. coli* HT115 (DE3) strain carrying vector L4440 express dsRNA corresponding to targeted genes to perform double-stranded (ds)RNA-mediated RNA interference (RNAi). As experimental control, *E. coli* HT115 carrying empty vector (no dsRNA expression) were used, labelled as EV. All strains were grown overnight in LB medium with 100 μg/mL ampicillin at 37 °C. Cultured *E. coli* strains spread onto NGM plates contained 100 μg/mL ampicillin and 1 mM isopropyl 1-thio-β-D-galactopyranoside (IPTG). RNAi assay were performed according to pervious study [55], in brief, synchronized L1 larvae (Bristol N_2_ strain of *C. elegans*) fed on RNAi bacteria strains at 20 °C until reach to young adult. Then young adult worms were transferred on the BHI plates for further studies.

### 4.3. C. elegans Survival Assays

The *C. elegans* strains were cultured on NGM plate with *E. coli* OP50 at 20 °C following the standard maintenance procedures [52]. Worms were synchronized by alkaline hypochlorite solution [56], eggs were incubated in M9 buffer at 20 °C, overnight for hatching. Hatched L1 larvae were transferred onto NGM plates seeded with live *E. coli* OP50 for further assays. To examine the effect of *N. farcinica* on worms’ lifespan, synchronized L1 larvae were cultivated fed *E. coli* OP50 at 20 °C until the young adult stage. Then 50–60 worms were fed with either *N. farcinica* or laboratory standard diet *E. coli* OP50 on BHI medium containing 5′-fluoro- 2′-deoxyuridine (FUdR) (75 μg/mL) at 25 °C. The survival curves were plotted by scoring the dead worm at 24 h interval. Immobile worms unresponsive to touch were scored as dead. Three plates were analyzed per assay and all experiments were performed three times. 

### 4.4. SKN-1 Nuclear Localization Assays

After young adult worms expressing *skn-1b/c::gfp* were fed with either *N. farcinica* or laboratory standard diet *E. coli* OP50 on BHI plates for 24 hours. Briefly, worms were mounted on slides from plates and imaged by using Nikon e800 fluorescence microscope. At least 30 worms were examined under each condition in three independent experiments.

### 4.5. Western Blot

After washed with M9 buffer, worms were homogenized in liquid nitrogen. Then the homogenate was lysed on ice for 30 min in lysis buffer RIPA (Beyotime Institute of Biotechnology, Haimen, China). After centrifuged at 12,000 rpm for 15 min at 4 °C, the supernatant was obtained and used for Western blot analysis. The total protein extraction was loaded on 10% SDS-PAGE for electrophoresis. Proteins were then transferred to immobilon-PSQ transfer PVDF membrane (Millipore, Bedford, MA, USA). Primary antibodies were anti-phospho-p38 antibodies (1:1000 dilution; Promega Biotech Co.,Ltd, Beijing, China), and anti-α-tubulin antibodies (1:1000 dilution; Abcam, Cambridge, UK). The secondary antibodies were peroxidase-coupled anti-rabbit IgG (1:10,000 dilution; Abmart, Shanghai, China). Blots were developed using Super Signal chemiluminescence substrate (Thermo Fisher Scientific, Waltham, MA). An imaging system (Amersham Imager 600) was used for documentation of the Western blot results. Band intensities were measured using ImageJ software (NIH).

### 4.6. Quantitative PCR Analysis

Total RNA was isolated from worms with TRIzol Reagent (Invitrogen, Carlsbad, CA). Random-primed cDNAs were generated by reverse transcription of the total RNA samples with SuperScript II (Invitrogen). A real time-PCR analysis was conducted using SYBR^®^ Premix-Ex TagTM (Takara, Dalian, China) on a Roche LightCycler 480^®^ System (Roche Applied Science, Penzberg, Germany). *act-1* gene was used for an internal control. The primers used for PCR are listed in Table S2.

### 4.7. Fluorescence Microscopic Analysis

For detecting fluorescence in worms, analysis, synchronized young adult worms expressing either *nlp-29p::gfp* or *gst-4p::gfp* were exposed to *N. farcinica* or *E. coli* OP50 for 24 h. Then the worms were mounted in M9 onto microscope slides. The slides were imaged using a Nikon e800 fluorescence microscope. Fluorescence intensity was quantified by using the ImageJ software (NIH). Mean value and standard errors were calculated based on more than 30 worms under each condition in three independent experiments.

### 4.8. ROS Analysis

ROS was detected by transferring worms in to M9 buffer with DHE (3 μM) and stained for 3 h before mounting in M9 buffer onto microscope slide, examined by fluorescence microscope (Zeiss Axioskop 2 Plus) [57].

### 4.9. The Colony Forming Units (CFU) Analysis

To ask if *N. farcinica* colonized in worms, we fed young adult worms with either *E. coli* OP50 or *N. farcinica* for 96 h on BHI plates, then worms were transferred in to M9 buffer containing 25 mM levamisole hydrochloride (Sangon Biotech Co., Shanghai, China), 50 μg/mL kanamycin and 100 μg/mL carbenicillin ( Sangon Biotech Co., Shanghai, China), and soaking for 30 min before washing with M9 buffer for three times. Worms were collected and grinded in PBS with 0.1% Triton, and serial diluted before placing on BHI ager for incubation, then bacterial colonies were counted to measure CFU.

### 4.10. Statistical Analysis

The statistical significance of differences in gene expression and fluorescence intensity was assessed by performing a one-way ANOVA followed by a Student-Newman-Keuls test and two-tailed unpaired *t*-test. Differences in survival rates were analyzed using the log-rank test. Difference of CFU counting was assessed by two-tailed unpaired t-test. Data were analyzed using SPSSsoftware, version 26.0 (SPSS Inc., Chicago, Illinois, USA) and Graphpad Prism 8.

## Data Availability

Not applicable.

## Supplementary Materials

The following supporting information can be downloaded at: https://www.mdpi.com/article/10.3390/pathogens11101071/s1, Table S1: Lifespan assay, Table S2: Primers of QPCR analysis.
