# Supplementary material for: The p38 MAPK/PMK-1 Pathway Is Required for Resistance to Nocardia farcinica Infection in Caenorhabditis elegance
Source: Pathogens. 2022 Sep 21;11(10):1071. doi: 10.3390/pathogens11101071 (PMC9609018; doi:10.3390/pathogens11101071)
Supplement: Supplementary file 1 [file pathogens-11-01071-s001.zip › Table S1ú║Lifespan assays.pdf]

Lifespan assays data

Genotype/condition	Median survival±Std. error	P value	N	Fig
N_2+OP50	18±1	--	58	Fig1
N_2+N.farcinica	18±0.9	0.2	71	Fig1
WT+OP50	19±0.5	--	63	Fig2.A
jnk-1(gk7)+OP50	17±0.9	0.0003	74	Fig2.A
WT+NF	18±0.5	--	73	Fig2.A
jnk-1(gk7)+NF	17±0.5	< 0.0001	54	Fig2.A
WT+OP50	19±0.5	--	55	Fig2.B
mpk-1(n2521)+OP50	18±0.8	0.0017	70	Fig2.B
WT+NF	19±0.9	--	58	Fig2.B
mpk-1(n2521)+NF	17±0.8	< 0.0001	68	Fig2.B
WT+OP50	19±0.8	--	50	Fig2.C
hlh-30(tm1978)+OP50	17±0.5	<0.0001	76	Fig2.C
WT+NF	19±0.9	--	57	Fig2.C
hlh-30(tm1978)+NF	17±0.5	< 0.0001	68	Fig2.C
Vector RNAi+OP50	20±1.24	--	54	Fig2.D
dkf-2 RNAi+ OP50	18±0.8	0.0010	45	Fig2.D
Vector RNAi+NF	19±0.4	--	58	Fig2.D
dkf-2 RNAi+ NF	17±0.9	< 0.0001	44	Fig2.D
Vector RNAi+OP50	19±0.5	--	56	Fig2.E
*fshr-1*RNAi+OP50	19±0.9	0.0696	56	Fig2.E
Vector RNAi+NF	19±0.8	--	62	Fig2.E
*fshr-1*RNAi+NF	18±0.5	0.0094	70	Fig2.E
Vector RNAi+OP50	20±0.5	--	62	Fig2.F
egl-30 RNAi+OP50	20±0.9	0.0718	43	Fig2.F
Vector RNAi+NF	19±1.4	--	54	Fig2.F
egl-30 RNAi+NF	18±0.8	< 0.0001	65	Fig2.F
Vector RNAi+OP50	19±0.8	--	49	Fig2.G
daf-16 RNAi+OP50	18±0.4	0.0006	61	Fig2.G
Vector RNAi+NF	19±1.2	--	55	Fig2.G
daf-16 RNAi+NF	17±1.2	< 0.0001	72	Fig2.G
WT+OP50	19±0.4	--	63	Fig2.H
pmk-1(km25)+OP50	17±0.9	<0.0001	69	Fig2.H
WT+NF	18±1.6	--	73	Fig2.H

pmk-1(km25)+NF	7±0.5	< 0.0001	51	Fig2.H
WT+OP50	19±0.5	--	73	Fig2.I
nsy-1(ag3)+OP50	17±0.5	< 0.0001	59	Fig2.I
WT+NF	20±0.8	--	54	Fig2.I
nsy-1(ag3)+NF	8±0.8	< 0.0001	58	Fig2.I
WT+OP50	20±0.5	--	69	Fig2.J
sek-1(ag1)+OP50	18±0.9	< 0.0001	75	Fig2.J
WT+NF	20±0.8	--	48	Fig2.J
sek-1(ag1)+NF	7±1.2	< 0.0001	61	Fig2.J
WT+OP50	19±1.4	--	63	Fig5.A
WT+NF	18±0.5	0.039	73	Fig5.A
skn-1(tm3411)+OP50	17±0.9	--	50	Fig5.A
skn-1(tm3411)+NF	7±0.9	< 0.0001	59	Fig5.A
WT+vector RNAi	18±0.5	--	53	Fig5.B
pmk-1(km25)+vector RNAi	7±0.9	< 0.0001	60	Fig5.B
pmk-1(km25)+skn-1 RNAi	6±1.7	< 0.0001	52	Fig5.B
