# Supplementary material for: The p38 MAPK/PMK-1 Pathway Is Required for Resistance to Nocardia farcinica Infection in Caenorhabditis elegance
Source: Pathogens. 2022 Sep 21;11(10):1071. doi: 10.3390/pathogens11101071 (PMC9609018; doi:10.3390/pathogens11101071)
Supplement: Supplementary file 1 [file pathogens-11-01071-s001.zip › Table S2ú║ Primers of QPCR analysis.pdf]

Table S2 Primers of QPCR analysis

Primer	Sequence (5’ to 3’)	Source or reference
Nlp-29-F<br>Nlp-29-R	TGTTCTTGTCGTCCTTCTCG<br>CCATAGCCTCCATAC ATTCCTC	This study
F08g5.6-F<br>F08g5.6-R	TGTGGC AAAGGAGAGGATG<br>ACGGTAGATTGCTAATGGGTTC	This study
F35e12.5-F<br>F35e12.5-R	ACTGTCAGAGCAGCAAATAACG<br>AAAGCGGTGTAATCAGGTCC	This study
Y37a1a.2-F<br>Y37a1a.2-R	TTCTGTCGTCTGGATTGGC<br>CGAGAGATA ACGGCTTGGTG	This study
Gst-4-F<br>Gst-4-R	TTGATGCTCGTGCTCTTGC<br>CAA ATGGAGTCGTTGGCTTC	This study
Act-1-F<br>Act-1-R	CCAATCCAAGAGAGGTATCC<br>GGCTTCAGTGAGGAGGAC	This study

The primers were designed in this study verified by PCR amplification before QPCR assay
